# Genetic Variants in *WNT16* and *PKD2L1* Locus Affect Heel Ultrasound Bone Stiffness: Analyses from the General Population and Patients Evaluated for Osteoporosis

**DOI:** 10.1007/s00223-023-01141-9

**Published:** 2023-10-13

**Authors:** Angelique Kragl, Anke Hannemann, Matthias Nauck, Uwe Völker, Heide Siggelkow, Alexander Teumer, Mladen V. Tzvetkov

**Affiliations:** 1 Institute of Pharmacology, Center of Drug Absorption and Transport (C_DAT), University Medicine Greifswald, Greifswald, Germany; 2 Institute of Clinical Chemistry and Laboratory Medicine, University Medicine Greifswald, Greifswald, Germany; 3 DZHK (German Centre for Cardiovascular Research), Partner Site Greifswald, University Medicine, Greifswald, Germany; 4 Interfaculty Institute of Genetics and Functional Genomics, Department Functional Genomics, University Medicine Greifswald, Greifswald, Germany; 5 MVZ Endokrinologikum Goettingen, Goettingen, Germany; 6 Clinic of Gastroenterology, Gastrointestinal Oncology and Endocrinology, University Medical Center Goettingen, Goettingen, Germany; 7 Institute for Community Medicine, University Medicine Greifswald, Greifswald, Germany; 8 Department of Psychiatry and Psychotherapy, University Medicine Greifswald, Greifswald, Germany

**Keywords:** Single nucleotide polymorphisms, SHIP, BMD, GWAS

## Abstract

Osteoporosis, a complex chronic disease with increasing prevalence, is characterised by reduced bone mineral density (BMD) and increased fracture risk. The high heritability of BMD suggests substantial impact of the individual genetic disposition on bone phenotypes and the development of osteoporosis. In the past years, genome-wide association studies (GWAS) identified hundreds of genetic variants associated with BMD or osteoporosis. Here, we analysed 1103 single nucleotide polymorphisms (SNPs), previously identified as associated with estimated BMD (eBMD) in the UK Biobank. We assessed whether these SNPs are related to heel stiffness index obtained by quantitative ultrasound in 5665 adult participants of the Study of Health in Pomerania (SHIP). We confirmed 45 significant associations after correction for multiple testing. Next, we analysed six selected SNPs in 631 patients evaluated for osteoporosis [rs2707518 (*CPED1/WNT16*), rs3779381 (*WNT16*), rs115242848 (*LOC101927709/EN1*), rs10239787 (*JAZF1*), rs603424 (*PKD2L1*) and rs6968704 (*JAZF1*)]. Differences in minor allele frequencies (MAF) of rs2707518 and rs3779381 between SHIP participants (higher MAF) and patients evaluated for osteoporosis (lower MAF) indicated a protective effect of the minor allele on bone integrity. In contrast, differences in MAF of rs603424 indicated a harmful effect. Co-localisation analyses indicated that the rs603424 effect may be mediated via *stearoyl-CoA desaturase* (*SCD*) expression, an enzyme highly expressed in adipose tissue with a crucial role in lipogenesis. Taken together, our results support the role of the WNT16 pathway in the regulation of bone properties and indicate a novel causal role of *SCD* expression in adipose tissue on bone integrity.

**Supplementary Information:**

The online version contains supplementary material available at 10.1007/s00223-023-01141-9.

## Introduction

In 2019, 32 million Europeans were estimated to have osteoporosis with the highest number of affected persons being estimated for Germany [1]. Osteoporosis is marked by reduced bone mineral density (BMD) and an increased risk of fractures. Affected patients suffer from increased morbidity and mortality [2] which is accompanied by reduced quality of life [3].

According to the World Health Organization (WHO), osteoporosis is defined by a dual X-ray absorptiometry (DXA) T-score of −2.5 or lower [4]. German [5] and international guidelines [1], therefore, recommend DXA measurements for diagnosis of osteoporosis. An alternative method to BMD measurement by DXA is the quantitative ultrasound (QUS) technique. Although QUS measurements are not directly comparable to DXA, they are free of ionising radiation, allow assessing physical bone properties, and predict fracture risk at different sites [6].

Osteoporosis risk is determined by both, genetic and non-genetic, factors. Non-genetic factors include lifestyle-related conditions like being underweight and smoking as well as unmodifiable factors including age and intake of certain medications [7]. A parental history of hip fracture is another important risk factor that points to the impact of the individual genetic disposition to bone phenotypes [8]. Indeed, not only BMD (h^2^ = 50–80%) but also ultrasound bone properties (h^2^ = 40–50%) possess a high heritability (h^2^) [8]. This resulted in efforts to find genetic variants contributing to BMD, bone microarchitecture, and fractures [8]. Causative mutations for rare but severe bone diseases such as osteogenesis imperfecta and sclerosteosis were successfully identified [9]. These important discoveries not only broaden our understanding of the physiology of bone but may also contribute to the identification of new pharmacotherapeutic targets [10].

Next to monogenic mutations causing rare diseases, it is of high interest to assess common genetic variants that contribute to the disease risk in the general population. Genome-wide association studies (GWAS) in large samples provide a powerful tool for the identification of disease-related genes in complex and highly prevalent diseases like osteoporosis [8]. So far, several GWAS have been performed to identify genes associated with BMD, QUS measures, bone microarchitecture, and fractures to finally trace genetic variants involved in the development of osteoporosis (for a review see [11]). Meta-analyses of GWAS identified and replicated several loci associated with BMD including single nucleotide polymorphisms (SNPs) related to *WNT16, RSPO3, TMEM135,* and many more [12]. In the latest and largest GWAS in the field, including more than 426,000 individuals from the UK Biobank, Morris and Kemp et al. [13] identified 1103 conditional independent SNPs associated with QUS-derived estimated BMD (eBMD) at a genome-wide significant level. These were mapped to 518 loci, among which *DAAM2* was selected, in-depth characterised, and suggested as a promising target for further investigation [13].

Here, we followed up on the results of Morris and Kemp et al. [13]. We assessed the associations of the 1103 SNPs with the QUS-derived stiffness index in two cohorts of the population-based Study of Health in Pomerania (SHIP). We aimed to examine which associations replicate in our precisely phenotyped population. Moreover, we investigated our results in two cohorts of patients evaluated for osteoporosis with DXA measurements at the spine and femoral neck.

## Materials and Methods

### Study Populations

#### SHIP

SHIP was established to collect and analyse data on health and disease in Northeast Germany. It consists of two non-overlapping, population-based cohorts, SHIP-START and SHIP-TREND. Both cohorts are based on representative samples of the adult inhabitants of the study region. Details on study design and sampling can be found elsewhere [14]. The study was approved by the ethics committee of the University of Greifswald and is conducted in line with the Declaration of Helsinki including obtainment of written informed consent from all participants.

In the present study, data from the second follow-up of the SHIP-START cohort (SHIP-START-2, *n* = 2333) and the baseline examination of the SHIP-TREND cohort (SHIP-TREND-0, *n* = 4420) were analysed as only in these study waves, quantitative ultrasound measurements (QUS) at the heel were performed. Data collection in SHIP-START-2 and SHIP-TREND-0 was conducted in parallel between 2008 and 2012 with similar methods and protocols [14]. From the total of 6753 SHIP-START-2 and SHIP-TREND-0 participants, we excluded all subjects with missing QUS or genotyping data, and all subjects that were treated with systemic glucocorticoids (ATC classification: H02AB), bisphosphonates (ATC classification: M05BA, M05BB), or other drugs affecting bone structure and mineralisation (ATC classification: M05BX). The final study population comprised 2108 SHIP-START-2 and 3557 SHIP-TREND-0 participants.

All SHIP participants in both cohorts underwent an extensive computer-assisted personal interview on lifestyle, medical history, and socio-demographic characteristics, and a large range of medical tests (for details see [14]). Standardised measurements of body height and weight were performed with calibrated scales, and body mass index (BMI) was calculated as weight (kg)/height^2^ (m^2^). All participants were offered whole body magnetic resonance imaging (MRI). From the images, the amount of abdominal subcutaneous (SAT) and visceral (VAT) adipose tissue was quantified [15]. Women aged 60 years or older and women aged between 40 and 60 years without menstrual cycling were classified as postmenopausal, all further women as premenopausal. Regular medication intake was categorised according to the anatomical-therapeutic-chemical (ATC) classification system. Information on secondary causes of osteoporosis was not collected.

#### OsteoGene

OsteoGene (DRKS ID: DRKS00016601) is a prospective study recruiting patients evaluated for osteoporosis at the community health centre *MVZ endokrinologikum Göttingen* (Germany). Enrolled patients were aged between 18 and 88 years and had a 20% increased 10-year fracture risk for vertebral or hip fractures. According to German guidelines [5], these patients underwent several diagnostic measures including DXA measurements. 98.3% of the patients were therapy naïve. Intake of inhalative or oral glucocorticoids was defined as exclusion criteria. In contrast, intake of calcium or vitamin D supplements, or hormone replacement therapy was no exclusion criteria. Additionally, information on secondary causes of osteoporosis was collected. The final study population included 232 patients that were recruited between December 2017 and October 2020. The study was approved by the Ethics Review Committee of the University Medical Center Göttingen. All participants provided written informed consent.

#### HSD

HSD was performed retrospectively in 452 German subjects. In short, patients that were to be evaluated for osteoporosis in the endocrine outpatient clinic of the University Medical Center in Göttingen were enrolled. The study population included in the present analyses comprised 399 patients with complete information on age and information on BMD of at least one location (spine, femoral neck). Additionally, information on secondary causes of osteoporosis was collected. Further details on the HSD cohort have been published previously [16].

### Assessment of Bone Properties and Fractures

#### SHIP

QUS measurements were performed at the heel of both feet using an Achilles InSight System (GE Medical Systems Ultrasound, GE Healthcare, Chalfont St Giles, UK). In short, two ultrasound parameters, the broadband ultrasound attenuation (BUA) and the speed of sound (SOS), were measured. These measures were combined to form the stiffness index (SI) according to the following formula: SI = (0.67 × BUA) + (0.28 × SOS)-420. The stiffness index serves as an indicator of the osteoporotic fracture risk. Statistical analyses were performed with data from the foot with the lower stiffness index. QUS measurements were not performed when the participant had implants, prostheses, or amputations in or below the knee, wounds, or infections distal to the knee, or oedema. Data from participants who reported an injury or surgery below the knee within twelve months prior to the measurement, who used a wheelchair or could not correctly place the feet into the device, were excluded from the statistical analyses. Data on self-reported incident fractures since the baseline examination were collected in SHIP-START-2 and data on selected lifetime fractures (proximal humerus, vertebral, hip, or femoral neck fractures) were collected in SHIP-TREND-0.

#### OsteoGene

Areal BMD (g/cm^2^) was measured by dual-energy X-ray absorptiometry (DXA) at the lumbar spine (L1-L4), total femur, and femoral neck of both legs using a LUNAR Prodigy instrument (GE Healthcare, Chicago, IL, USA). T-Score and Z-Score were automatically determined by the instrument. For analyses, no less than two vertebrae and only vertebrae without fractures were included. Vertebral fractures were also assessed by DXA scan. Peripheral fracture rate was assessed by already available X-ray or MRI scans.

#### HSD

BMD was determined by DXA measurements at the lumbar spine and the left femoral neck. Fractures were self-reported and partially cross-checked against radiology reports and fracture clinic attendance.

### Genotyping

#### SHIP

SHIP-START participants were genotyped applying the Affymetrix Genome-Wide Human SNP Array 6.0 (Santa Clara, CA, USA). SHIP-TREND-0 participants were genotyped applying either the Illumina Infinium® HumanOmni2.5 BeadChip or the Illumina Infinium® Global Screening Array (San Diego, CA, USA). Genotyping was performed according to the manufacturer’s protocol. Whole-genome imputation was performed on the Michigan Imputation Server using the HRC reference panel (version r1.1 2016).

#### OsteoGene and HSD

DNA was isolated from blood samples with the QIAamp DNA Blood Mini Kit (Qiagen, Hilden, Germany). The rhAmp SNP Genotyping System (Integrated DNA Technologies, Carolville, IA, USA) was used to genotype rs2707518 (*CPED1*/*WNT16*; Assay ID: CD.GT.FSGQ5187.1), rs3779381 (*WNT16*; CD.GT.PBLY8533.1), rs603424 (*PKD2L1*; Hs.GT.rs603424.A.1), rs10239787 (*JAZF1*; Hs.GT.rs10239787.T.1), and rs6968704 (*JAZF1*; Hs.GT.rs6968704.T.1) in 5 µl reactions in 384-well plates according to the manufacturer’s protocol but using undiluted DNA. The PCR, data collection, and analysis were conducted in a QuantStudio 12k Flex Real-Time PCR System (Thermo Fisher Scientific, Waltham, MA, USA).

### Statistical analyses

Characteristics of the SHIP participants and the patients evaluated for osteoporosis are reported as means with standard deviation or proportions.

In SHIP, associations between the SNPs and stiffness index were determined separately for the two cohorts using multivariate linear regression models implemented in EPACTS version 3.2.6 patched (http://csg.sph.umich.edu//kang/epacts/download/). Sex and age were defined as covariates. As genotyping in the SHIP-TREND cohort was performed with two arrays, three further covariates were defined for this cohort: genotyping array and the first two genetic principal components. The individual results were combined by fixed effects inverse-variance weighted meta-analysis using METAL [17]. The false discovery rate (FDR) at 5% using the Benjamini–Hochberg procedure was calculated to account for multiple testing [18]. Results were called significant when the FDR was < 0.05. We report effect estimates with standard error, p-value, and FDR from these models. The results of the meta-analyses were further illustrated in a plot depicting the absolute effect size in relation to the minor allele frequency (MAF).

We then selected the five SNPs with the lowest p-values from the meta-analysis for genotyping in the OsteoGene and HSD study cohorts: rs2707518, rs3779381, rs115242848, rs10239787, and rs603424. These SNPs are located in *CPED1/WNT16*, *WNT16*, *LOC101927709/ EN1*, *JAZF1,* and *PKD2L1*, respectively. In addition, rs6968704 (*JAZF1*) which was also significantly associated with stiffness index, was selected for genotyping. Thus, a total of six SNPs were genotyped in the patient cohorts. Associations between the SNPs and BMD at the femoral neck or spine were assessed with linear regression models adjusted for sex and age (IBM SPSS Statistics v.26, IBM, Armonk, NY, USA). Next to a combined model with pooled data from OsteoGene and HSD, also separate models for the two cohorts were calculated. Finally, we compared the MAFs of the six selected SNPs between SHIP participants and patients evaluated for osteoporosis.

Linkage disequilibrium was analysed using SNiPA [19] with the following settings: Genome assembly: GRCh37, Variant set: 1000 Genomes, Phase 3 v5, Population: European. Data plotting was performed with GraphPad Prism v.5.01.

Co-localisation analyses were conducted to assess effects of genetically predicted gene expression mRNA levels from 49 tissues obtained via eQTLs from the GTEx v8 database (EUR sample, https://gtexportal.org/) on stiffness index. To increase the robustness of these analyses, two different co-localisation methods were applied, focussing on the intersection of the significant results.

For both methods, the associations with the stiffness index of all SNPs within 1.1 Mb around rs603424, as well as the eQTLs of the corresponding regions per tissue, were extracted. First, Bayesian co-localisation analyses were conducted using the R-package “gtx” version 2.1.6 (https://github.com/tobyjohnson/gtx, ‘coloc.fast’ function with 100 kb SNP window and default parameters and prior definitions), which implemented the co-localisation method of Giambartolomei et al. [20]. For all co-localisation analyses, a posterior probability (PP) of ≥ 0.80 of the H4 test (both trait and expression data are associated and share the same single causal variant) was applied to identify significant results.

Second, the SNP rs603424 was tested and plotted for co-localisation with the tissue-specific mRNA levels by applying the summary-data-based Mendelian randomisation (SMR) method [21]. The method includes a test whether the effect on expression observed at a SNP is independent of the signal observed in the trait association (SMR test) and a second test that evaluates if the eQTL and trait associations can be attributable to the same causative variant by performing a heterogeneity test (HEIDI test). Significance for co-localisation of the gene expression and the trait signals was defined by p_SMR_ < 0.001, where additionally a p_HEIDI_ ≥ 0.05 indicates the same underlying causal variant.

Finally, we assessed the associations between rs603424 and the amount of SAT, VAT, and the ratio of VAT/SAT in SHIP-START-2 and SHIP-TREND-0. Cohort-specific linear regression analyses with log-transformed adipose tissue markers as outcome and rs603424 as exposure were calculated. The adjustment of the models followed the adjustment in the genome-wide association study. Following this, the results were combined by a fixed effects inverse-variance weighted meta-analysis analogue to the GWAS meta-analysis.

## Results

General characteristics of the SHIP participants and the HSD and OsteoGene patients are listed in Table 1. In the two patient cohorts, women were overrepresented, while the sex ratio was balanced in the SHIP cohorts. Patients in the OsteoGene cohort were older (average age 66.0 years) than patients in the HSD cohort (56.2 years), in SHIP-START-2 (56.8 years) and SHIP-TREND-0 (50.9 years). Among HSD and OsteoGene patients, secondary osteoporosis was diagnosed in 48.9% and 51.3%, respectively. Moreover, patients evaluated for osteoporosis had on average a lower BMI than SHIP participants. Fractures were reported by less than 10% and intake of vitamin D or calcium supplements by less than 2% of SHIP participants. These values were expectedly higher in the patients (Table 1). The QUS-based stiffness index was comparable between SHIP-START-2 and SHIP-TREND-0, while BMD at the spine and femoral neck was lower in patients from the HSD cohort than in OsteoGene patients.

**Table 1:** Characteristics of SHIP participants and patients evaluated for osteoporosis

Characteristics	General population		Patients evaluated for osteoporosis	
	**SHIP-START-2** (*n* = 2108)	**SHIP-TREND-0** (*n* = 3557)	**HSD** (*n* = 399)	**OsteoGene** (*n* = 232)
Women, %	52.6	50.7	72.4	83.6
Age, years	56.8 (13.5)	50.9 (15.0)	56.2 (14.1)	66.0 (11.0)
BMI, kg/m^2^	28.2 (4.84)	27.9 (5.12)	25.1 (4.52)	24.7 (4.57)
Menopausal status (in women)				
Premenopausal, %	32.4	46.0	28.4	3.60
Postmenopausal, %	67.6	54.0	68.2	96.4
Calcium supplements, %	1.42	0.00	60.2	12.9
Vitamin D supplements, %	1.42	1.21	50.4	71.1
Oral contraceptives (in premenopausal women), %	25.1	30.4	48.8	n.a
Secondary osteoporosis, %	–	–	48.9	51.3
Fractures, % Peripheral, % Spine, %	9.87* – –	5.78* – –	36.6 17.5 23.6	52.6 39.2 19.4
Stiffness index	92.1 (18.0)	94.4 (18.2)	–	–
BMD spine, g/cm^2^	–	–	0.83 (0.16)	0.93 (0.16)
BMD, femoral neck, g/cm^2^	–	–	0.70 (0.12)	0.77 (0.12)

Data are proportions or mean (standard deviation)

*Self-reported fractures in SHIP comprise selected lifetime fractures in SHIP-TREND-0 (proximal humerus, vertebral, hip, or femoral neck fractures) as well as any incident fracture since the baseline examination in SHIP-START-2

Missings in SHIP-START-2: fractures: 0.47%, menopausal status: 0.27%. Missings in SHIP-TREND-0: fractures: 0.31%, menopausal status: 0.06%. Missings in HSD: BMI: 0.8%; menopausal status: 3.5%; calcium supplements: 1.5%; vitamin D supplements: 1.5%; oral contraceptives: 31.3%; secondary osteoporosis: 10.8%; fractures: 0.8%; fractures, peripheral: 1.3%; fractures, spine: 0.8%; BMD spine: 4.2%; BMD femoral neck: 4.8%. Missings in OsteoGene: fractures, peripheral: 0.4%; fractures, spine: 8.6%; BMD spine: 0.9%; BMD femoral neck: 1.8%

*BMI* body mass index, *BMD* bone mineral density

The associations between the 1103 SNPs reported by Morris and Kemp et al. [13] and the QUS-based stiffness index were examined in the two SHIP cohorts, and the results were combined in a meta-analysis. This analysis, including 2108 SHIP-START-2 and 3557 SHIP-TREND-0 participants (total of 5665), yielded 45 significant associations after correction for multiple testing (Table 2). Among the associated SNPs, there were two in the *WNT16* locus (rs2707519 and rs3779381), as well as SNPs in *EN1*, *JAZF1*, *PKD2L1*, *SPTBN1*, *GPC6*, *TMEM135,* and further loci. From this list, we selected the five SNPs with the lowest p-values for further analyses in patients evaluated for osteoporosis: rs2707518 (*CPED1*/*WNT16*), rs3779381 (*WNT16*, intron 1), rs115242848 (*LOC101927709*/*EN1*), rs10239787 (*JAZF1*, intron 2), and rs603424 (*PKD2L1*, intron 2). We additionally chose rs6968704 (*JAZF1*, intron 2) to receive more information about the *JAZF1* locus.

**Table 2:** SNPs associated with stiffness index after correction for multiple testing. Results from the meta-analysis combining SHIP-START-2 and SHIP-TREND-0 (*n* = 5665)

dbSNP ID	Position	Genetic variants	Genetic location	MAF	Effect size *	Stderr	p	FDR
**rs2707518**	chr7:120,954,908	G > T	*CPED1/WNT16*	0.372	+2.327	0.331	2.03E-12	2.24E-09
**rs3779381**	chr7:120,966,790	A > G	*WNT16*	0.240	+2.229	0.370	1.76E-09	9.73E-07
**rs115242848**	chr2:119,507,607	C > T	*LOC101927709/EN1*	0.013	+6.702	1.388	1.37E-06	5.06E-04
**rs10239787**	chr7:27,970,153	C > T	*JAZF1*	0.358	−1.587	0.337	2.50E-06	5.59E-04
**rs603424**	chr10:102,075,479	G > A	*PKD2L1*	0.178	−1.956	0.416	2.53E-06	5.59E-04
rs75475627	chr2:54,787,592	C > G	*SPTBN1*	0.063	−3.039	0.659	3.98E-06	7.32E-04
rs72640504	chr13:94,066,952	T > C	*GPC6*	0.008	−8.074	1.788	6.29E-06	9.93E-04
rs75937733	chr11:86,905,021	A > G	*TMEM135*	0.077	+2.519	0.607	3.30E-05	4.16E-03
rs2609352	chr1:223,184,621	C > A	*DISP1/TLR5*	0.318	−1.423	0.346	4.02E-05	4.16E-03
rs2929308	chr8:9,084,121	T > A	*LOC101929128/ LOC107986914*	0.472	−1.324	0.323	4.08E-05	4.16E-03
rs2971879	chr2:54,888,533	T > C	*SPTBN1*	0.243	+1.526	0.372	4.14E-05	4.16E-03
rs6932260	chr6:151,939,560	T > C	*CCDC170*	0.491	−1.304	0.321	4.85E-05	4.46E-03
**rs6968704**	chr7:27,966,984	C > T	*JAZF1*	0.302	+1.427	0.354	5.49E-05	4.67E-03
rs78438678	chr20:10,634,675	C > T	*JAG1*	0.077	−2.221	0.592	1.75E-04	1.38E-02
rs7209460	chr17:2,048,713	C > T	*SMG6*	0.329	+1.255	0.342	2.44E-04	1.69E-02
rs7167692	chr15:85,660,184	T > C	*PDE8A*	0.048	−2.726	0.747	2.62E-04	1.69E-02
rs2566774	chr1:68,694,877	T > C	*WLS*	0.191	−1.482	0.407	2.75E-04	1.69E-02
rs4233949	chr2:54,659,707	C > G	*LOC102724072*	0.394	−1.183	0.326	2.89E-04	1.69E-02
rs4635400	chr18:13,719,510	G > A	*FAM210A*	0.390	−1.202	0.332	2.91E-04	1.69E-02
rs947091	chr10:31,054,186	G > A	*LYZL2/ZNF438*	0.460	+1.153	0.320	3.18E-04	1.76E-02
rs28626308	chr19:33,517,515	C > T	*RHPN2*	0.057	+2.354	0.665	3.99E-04	1.87E-02
rs370387	chr3:41,123,984	G > A	*RPS27P4/CTNNB1*	0.442	+1.138	0.322	4.08E-04	1.87E-02
rs13267351	chr8:77,483,797	C > T	*LOC107986952*	0.153	+1.557	0.441	4.17E-04	1.87E-02
rs7488974	chr12:90,442,001	G > A	*LOC105369890*	0.389	+1.155	0.328	4.23E-04	1.87E-02
rs1897468	chr2:237,688,196	C > T	*LOC105373949*	0.283	−1.263	0.358	4.24E-04	1.87E-02
rs117111740	chr11:62,201,239	T > C	*AHNAK*	0.019	−4.230	1.209	4.67E-04	1.98E-02
rs144832051	chr2:119,610,406	C > T	*EN1/MARCO*	0.022	+3.819	1.111	5.86E-04	2.32E-02
rs7703751	chr5:122,831,981	A > T	*CEP120/CSNK1G3*	0.234	−1.287	0.374	5.88E-04	2.32E-02
rs11763267	chr7:20,052,860	T > C	*LOC101927668*	0.171	−1.465	0.430	6.64E-04	2.45E-02
rs9379084	chr6:7,231,843	G > A	*RREB1*	0.103	−1.747	0.513	6.65E-04	2.45E-02
rs2305489	chr4:997,488	G > T	*IDUA*	0.060	+2.391	0.706	7.08E-04	2.50E-02
rs2272224	chr7:96,308,943	T > C	*SEM1*	0.298	+1.192	0.353	7.25E-04	2.50E-02
rs482339	chr11:86,857,174	C > A	*TMEM135*	0.294	−1.190	0.354	7.74E-04	2.59E-02
rs3760456	chr17:27,948,844	C > T	*CORO6*	0.430	−1.069	0.325	1.00E-03	3.16E-02
rs1871859	chr6:151,898,506	C > T	*CCDC170*	0.131	−1.587	0.482	1.00E-03	3.16E-02
rs698891	chr1:16,274,769	C > T	*ZBTB17*	0.108	+1.693	0.517	1.05E-03	3.21E-02
rs9594738	chr13:42,952,145	C > T	*AKAP11/LINC02341*	0.478	−1.053	0.323	1.11E-03	3.21E-02
rs9606138	chr22:19,676,393	G > A	*LOC100420103/SEPTIN5*	0.105	−1.768	0.542	1.11E-03	3.21E-02
rs938295	chr1:16,087,260	C > T	*FBLIM1*	0.209	+1.277	0.392	1.13E-03	3.21E-02
rs7121746	chr11:112,437,007	A > G	*LINC02764/LOC124902757*	0.425	−1.049	0.324	1.20E-03	3.31E-02
rs12460389	chr19:33,569,747	T > A	*RHPN2/GPATCH1*	0.138	+1.476	0.461	1.36E-03	3.58E-02
rs7040344	chr9:133,452,158	C > T	*ASS1/LOC100272217*	0.331	−1.099	0.344	1.39E-03	3.58E-02
rs11880992	chr19:2,176,403	G > A	*DOT1L*	0.440	−1.038	0.325	1.39E-03	3.58E-02
rs1661725	chr17:73,560,134	T > C	*LLGL2*	0.393	+1.046	0.331	1.56E-03	3.83E-02
rs1622638	chr11:121,800,971	G > A	*SORL1/MIR100HG*	0.425	+1.034	0.327	1.56E-03	3.83E-02

Position based on GRCh37.p13; Genetic variants, major > minor allele, in the analysis the minor allele was the coded allele; MAF, minor allele frequency; Effect size, unstandardised regression coefficient; Stderr, standard error; p, significance; FDR, false discovery rate; *, + indicates that the minor allele increases the stiffness index,-indicates that the minor allele decreases the stiffness index; SNPs printed in bold were selected for genotyping in the HSD and OsteoGene cohorts

**Fig. 1:**
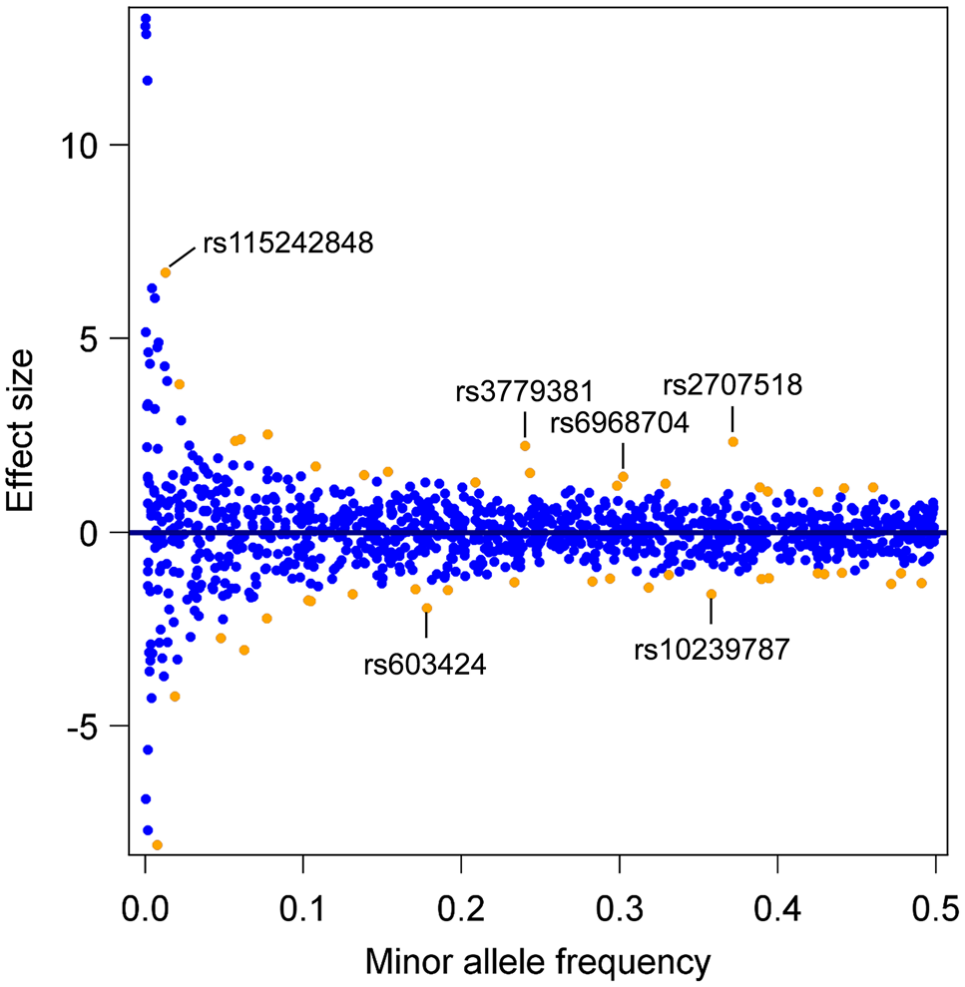
Effect size according to minor allele frequency (MAF) of all analysed 1103 SNPs in the meta-analysis (*n* = 5665 SHIP-START-2 and SHIP-TREND-0 participants). Orange dots represent SNPs that were significantly associated with the stiffness index after correction for multiple testing. Orange dots that were named represent the SNPs that were selected for further analysis in the patients evaluated for osteoporosis. Blue dots represent SNPs that were not significantly associated with the stiffness index

The meta-analysis combined the effect of the minor alleles of both SHIP cohorts of each of the 1,103 SNPs on the stiffness index. A positive effect size indicates an increase in stiffness index, and a negative effect size a decrease in stiffness index per minor allele. SNPs whose minor alleles were carried by less than 5% of the SHIP participants (MAF below 0.05) had the greatest effects on stiffness index (effect size below −3.0 or above +3.0), while SNPs with more frequent minor alleles had lower effects on stiffness index (MAF above 0.05 and effect sizes between −2.2 and +2.5) (Fig. 1). The selected SNPs (rs2707518, rs3779381, rs115242848, rs10239787, rs603424, rs6968704) had MAFs between 0.013 and 0.370 and effect sizes between −1.96 and +6.70 (Fig. 1, Table 1).

**Fig. 2:**
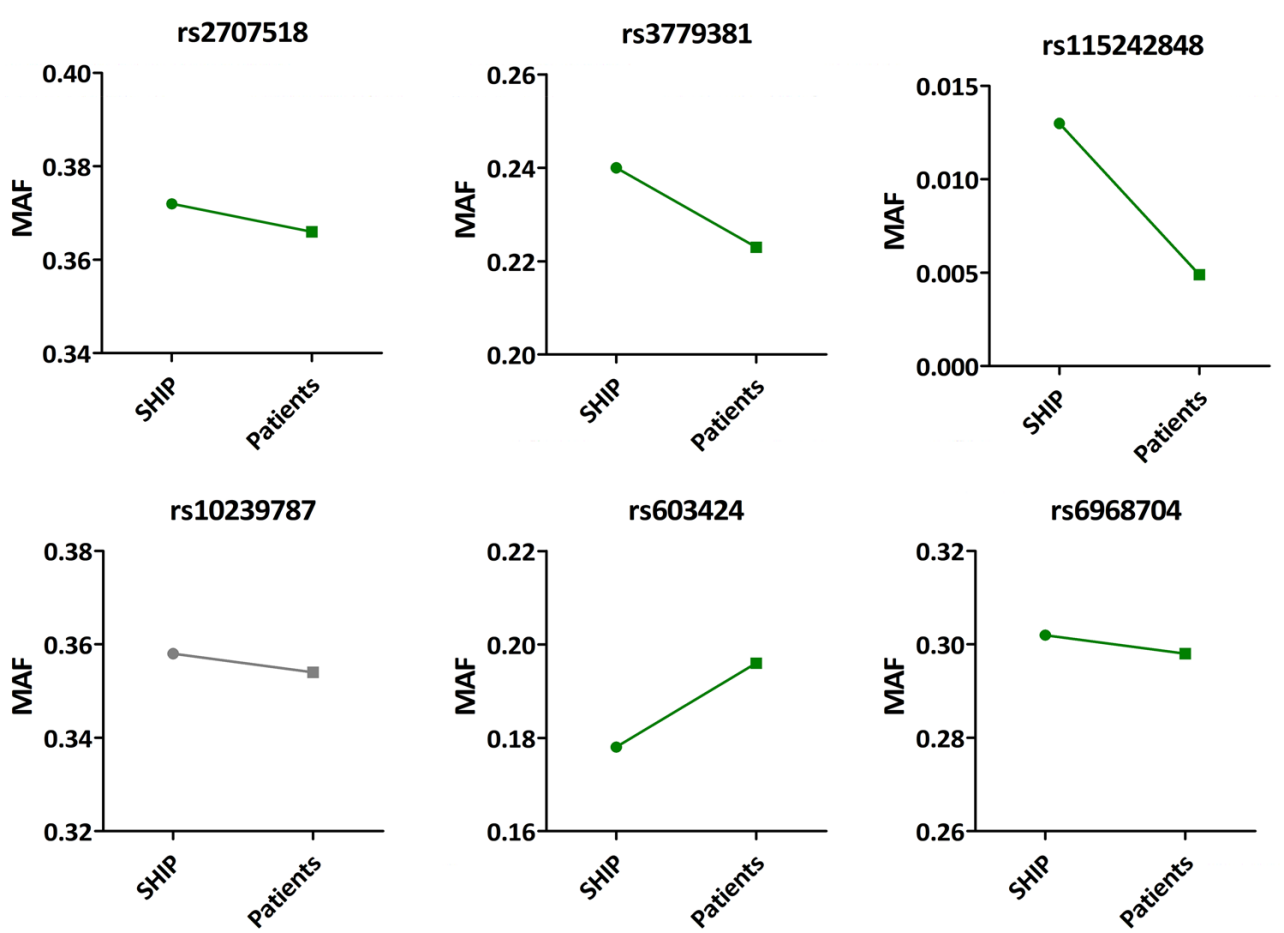
Comparison of the minor allele frequency (MAF) of the selected SNPs in the general population in SHIP-START-2 and SHIP-TREND-0 (denoted as SHIP), and the MAF of patients evaluated for osteoporosis in HSD and OsteoGene (denoted as patients). We hypothesised that a minor allele with protective effect on BMD (as inferred from the meta-analysis) is less represented in patients evaluated for osteoporosis than in individuals from the general population, whereas a minor allele with a negative effect on BMD is more represented. Green colour indicates confirmation of the hypothesis, grey colour indicates no confirmation

Subsequent analysis in patients of the HSD or OsteoGene studies revealed no statistically significant associations between the six genotyped SNPs and femoral neck or spine BMD. Also in analyses combining HSD and OsteoGene, no statistically significant results were obtained (Supplemental Table 1). Yet we observed trends toward differences in MAF between SHIP participants (representative of the general population) and the patients of the HSD and OsteoGene studies (Fig. 2 and Supplemental Table 2). While 24.0% of the SHIP participants carried the minor allele of rs3779381, there were only 22.9% minor allele carriers among the patients. The minor allele was positively associated with stiffness index in our meta-analysis [β =  +2.22, standard error (stderr) = 0.37] indicating a protective effect on BMD. Comparable observations were made for the protective minor alleles of rs2707518 and rs698704, although the differences in MAF between SHIP participants and patients evaluated for osteoporosis were below 0.3%. Also, rs115242848 was associated with an increased stiffness index in our meta-analysis. Its MAF was very low in SHIP (1.30%) but even lower in the patients (0.49%). Rs603424 on the other side was inversely associated with stiffness index (β = −1.96, stderr = 0.42), and fewer minor allele carriers were observed among the SHIP participants (17.8%) than among the HSD and OsteoGene patients (19.5%). Only rs10239787, which was inversely related to stiffness index (β = − 1.59, stderr = 0.42) had a higher MAF in SHIP participants (35.8%) than in the patients (35.4%). When assessing the two patient cohorts separately, we observed that all trends for differences in MAF were present in the OsteoGene cohort, while in the HSD cohort, only the results for rs3779381, rs115242848, and rs603424 were stable (Supplemental Table 2 and Supplemental Fig. 1).

**Fig. 3:**
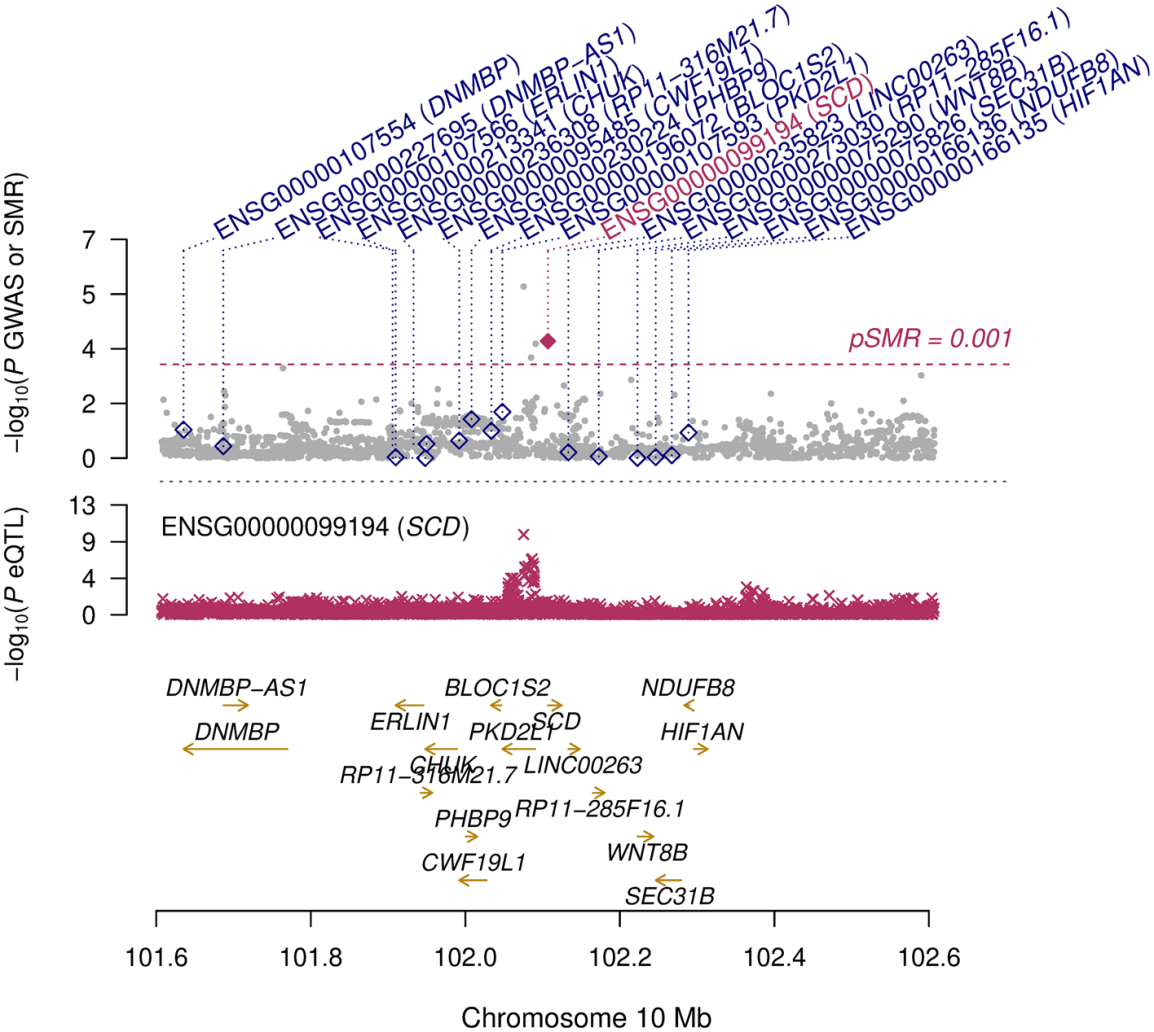
Co-localisation results. Illustration of the summary data-based Mendelian randomisation (SMR) test for the stiffness index and expression quantitative trait loci (eQTL) in adipose visceral tissue at the rs603424 locus. The upper box shows the regional association plot of the genetic associations with the stiffness index, with level of significance of the SMR test (y-axis) for each mRNA transcript in the locus indicated by a diamond positioned at the corresponding gene. A significant SMR test represented by a purple diamond indicates an association of the transcript level of the respective genes (purple label) with the trait. The filled purple diamond indicates a HEIDI test p-value > 0.05, thus, a likely co-localisation. The lower box shows the regional association distribution with changes in expression of the highlighted (purple) gene transcript. In both boxes, the x-axis refers to GRCh37/hg19 genomic coordinates

To test whether the genetic effect of a locus on BMD was mediated by gene expression of a nearby gene, we applied two co-localisation methods. These methods test for an association of gene expression and stiffness index estimated by SNP-mRNA and SNP-BMD association results. Both co-localisation methods indicated that higher mRNA expression of *stearoyl-CoA desaturase (SCD)* in visceral adipose tissue is associated a higher stiffness index (Fig. 3, Supplementary Tables 3 and 4). Our analyses further revealed that rs603424 affects *SCD* expression. Rs603424 is located in intron 2 of *PKD2L1*, a neighbouring gene of *SCD.* Its minor allele was associated with a decreased *SCD* mRNA expression in visceral adipose tissue. Thus, the minor allele of rs603424 may tag for effects on ultrasound-based heel stiffness index via changes in *SCD* gene expression. To follow-up on this, we examined whether rs603424 was associated with the amount of SAT, VAT or the ratio of VAT/SAT. Respective analyses yielded, however, no statistically significant results (Supplemental Table 5).

## Discussion

In the present analyses, we confirmed several loci associated with QUS-based bone properties in 5665 adults from the general population. Six of these SNPs, mapping to *WNT16*, *EN1*, *JAZF1,* and *PKD2L1*, were genotyped among 631 patients evaluated for osteoporosis. Tendencies towards differences in MAF between individuals from the general population and patients evaluated for osteoporosis support effects of five of these SNPs on bone substance. Moreover, co-localisation analyses pointed to a causal effect of gene expression of *SCD* on heel bone stiffness index (for a more detailed description of the respective genes see Supplemental Table 6).

While previous studies described in total more than a thousand genetic variants related to BMD [8, 11], eBMD [13], or fractures [22], it largely remains unclear what the causal mechanisms underlying the observed associations are. Methods to uncover causality include the identification and co-localisation of eQTL [23]. These approaches generate insights into the (patho-)physiology of bone metabolism. We complemented results obtained from a GWAS meta-analysis with eQTL co-localisation results and assessed MAFs of the promising variants in individuals from the general population and two cohorts of patients evaluated for osteoporosis. While the most prominent association with bone stiffness in our data was observed for two known SNPs in the *WNT16* locus, eQTL co-localisation analyses pointed to a causal role of rs603424 in bone integrity.

### WNT16 (rs2707518, rs3779381)

The Wnt-signalling pathway is crucial for the maintenance of bone homeostasis [24]. *WNT16* encodes Wnt-16 and is part of the *WNT* gene family, which was identified to positively regulate osteoblast [25] and negatively regulate osteoclast differentiation [26]. It is highly expressed in osteoblasts of cortical bone [26] and was shown to be crucial for the preservation of cortical [26] and trabecular bone mass [27]. Wnt-16 has been identified as a target of glucocorticoid action with Wnt-16 suppression resulting in decreased bone formation [28]. Genetic variations in *WNT16* were previously associated with BMD and fracture risk at genome-wide significance [29, 30]. Among these variants, two missense variants rs2707466 (Thr > Ile) and rs2908004 (Gly > Arg) were repeatedly shown to be related to BMD [31, 32].

In the present work, we focused on two non-coding SNPs, rs2707518 (*CPED1*/*WNT16*) and rs3779381 (*WNT16*), which were highly associated with eBMD in the UK Biobank [13] and with stiffness index in the SHIP cohorts. Rs2707518 is highly genetically linked (r^2^ = 0.99) to rs2536195 (*CPED1*/*WNT16*) which previously showed an association with stiffness index in a GWAS meta-analysis [33]. Rs3779381, in turn, was observed to be related to an increased osteoporosis risk in postmenopausal, overweight Chinese women [29]. Among the patients evaluated for osteoporosis, both SNPs demonstrated a lower MAF (0.366 for rs2707518 and 0.229 for rs3779381). The observed difference could, thus, represent an indicator of patient selection. Also, the effect direction observed in SHIP is in line with the results from the UK Biobank [13]. The minor T (rs2707518) and G (rs3779381) alleles were associated with increased stiffness index or eBMD. The minor alleles may, thus, confer a protective effect on bone properties, and carriers of these protective minor alleles may be less often found among patients than among the general population. Therefore, our data infer that these non-coding variants in the *WNT16* locus may play an important role in maintaining bone integrity.

### PKD2L1 (rs603424)

Morris and Kemp et al. [13] were the first to demonstrate a genome-wide significant association of rs603424 with adult eBMD. Their analyses suggest a potentially harmful effect of that SNP on eBMD [13], which was confirmed by our finding of an inverse association with the QUS-based stiffness index. The observation of a tendency towards fewer minor allele carriers among the SHIP participants (17.8%) than among the patients evaluated for osteoporosis (19.5%) further provides support for a potentially harmful effect of this SNP.

Co-localisation analyses suggest that rs603424 may impact on bone stiffness via modification of *stearoyl-CoA desaturase* (*SCD*) expression in adipose tissue. SCD is a key enzyme in lipogenesis that catalyses the synthesis of saturated fatty acids to monounsaturated fatty acids [34]. In humans, two SCD isoforms have been identified, SCD1 and SCD5. While SCD5 is mainly expressed in the brain and pancreas, SCD1 is more ubiquitously expressed, e.g. in adipose tissue, liver, brain, heart, and pancreas [34]. Over the last years, several studies reported SCD1 activity to be related to a disturbed lipid metabolism in obesity and non-alcoholic fatty liver disease as well as tumour malignancy [34, 35]. Yet the actions of SCD1 are complex and still not fully elucidated. Thus, SCD1 was described to exert beneficial effects by acting anti-inflammatory while inhibition of SCD1 increased saturated fatty acid levels and inflammation [36]. More important for the present work, SCD1 was reported to be involved in bone homeostasis, by promoting osteogenic differentiation of bone marrow mesenchymal stem cells [37]. In line with this, a connection between SCD and the Wnt pathway was identified as SCD1 and SCD2 were shown to provide a necessary lipid modification for Wnt biogenesis and pathway activation [38].

While our data point to an association of rs603424 with bone properties via affecting *SCD1* expression, an association between rs603424 and SAT, VAT or the ratio of VAT/SAT was not significant (all p-values > 0.58). However, it must be noted that only about half of the SHIP participants underwent MRI and provided data for the analyses, which strongly limits statistical power. Moreover, it might be awarding to assess further measures of quantity or quality of adipose tissue and to unravel mechanistic insights of SCD1. Our results presented here, will further need follow-up in functional analyses.

### EN1 (rs115242848)

Within our analyses, rs115242848 showed the largest effect on the stiffness index (β =  +6.702). This low-frequency SNP is an intergenic variant close to *EN1* which encodes the homeobox protein engrailed-1 [39]*.* Styrkarsdottir and colleagues [40] previously observed an association of rs115242848 with hip and spine BMD. In the SHIP data, the minor T allele of rs115242848 was associated with higher stiffness index values, indicating a protective effect on bone health. Quite similar, genome-wide significant effects on total body BMD were reported from the Life-Course GWAS meta-analysis [41]. Moreover, associations of rs115242848 with increased lumbar spine BMD [42], lumbar spine area [40], forearm and femoral neck BMD [42], and inverse associations with osteoporosis [43] and fractures [13] were reported but missed genome-wide significance. The MAF of rs115242848 was low among the SHIP participants (0.013) but higher than in other populations (0.010 in 1000G Europe, 0.008 in TWINSUK). The MAF in patients evaluated for osteoporosis (0.005) was even lower. Taking this low MAF and, thus, the low number of individuals carrying this variant in the HSD and OsteoGene cohort into account, no definite conclusions on the association of rs115242848 with BMD can be made.

### JAZF1 (rs10239787, rs6968704)

*JAZF1* encodes the transcriptional corepressor JAZF Zinc Finger 1 [44, 45]. Previously, associations of *JAZF1* with 124 different traits have been reported [46]. Among these, associations with anthropometric measures are dominating, but associations with diabetes, prostate cancer, and heel BMD have also been found [46]. The two, in our data, replicated SNPs had an opposite effect on the QUS-based stiffness index. While rs10239787 was related to lower values (β = −1.59), rs6968704 was associated with higher values (β =  +1.43). The potentially deleterious impact of rs10239787 on eBMD is in line with the results of Morris and Kemp et al. [13]. An inverse association of rs10239787, but without genome-wide significance, was further reported with total body BMD [41] and a corresponding positive association with fractures [47]. Regarding rs6968704, we are the first to independently confirm genome-wide significance with bone properties. Moreover, our results suggest a tendency towards higher MAF of the protective minor allele in the general population than in patients evaluated for osteoporosis. This implies that individuals lacking this protective variant may be overrepresented among the patients. This notion is, however, quite speculative, especially as previous studies demonstrated positive associations of rs6968704 with total body BMD [41] and inverse associations with lumbar spine area [48] but both without genome-wide significance.

## Strengths and Limitations

The present study stands out due to its sample of well-characterised individuals from a general population of European ancestry, and the simultaneous complementary evaluation of two samples of patients evaluated for osteoporosis. One limitation is that in the population-based SHIP cohorts, dual-energy X-ray absorptiometry (DXA) scans, using ionising radiation, were impossible due to ethical objections. This prohibited a direct comparison of BMD between SHIP participants and HSD or OsteoGene patients. Moreover, due to differences in bone structure throughout the skeleton, the QUS-based measurements at the heel are not directly comparable to measurements obtained at the femoral neck or spine. Nevertheless, QUS measurements allow osteoporotic fracture risk prediction [6]. The main results from our genetic analysis, i.e. the six selected SNPs, were fostered by the comparison of the MAFs of the minor alleles between SHIP participants and HSD and OsteoGene patients. Yet we cannot exclude that the use of the two methods to evaluate bone traits prevented a full confirmation in the patients evaluated for osteoporosis, i.e. significant associations in the regression models. We can further not rule out that differences in patient characteristics between HSD and OsteoGene contributed to more stable MAF differences in the OsteoGene cohort, than in the HSD cohort. Although there are differences, the majority of results for the HSD and OsteoGene cohorts points in the same direction and allows drawing similar conclusions. Above this, our study has the limitation that, in contrast to the study by Morris and Kemp et al. [13], it may not be large enough to estimate the effect of rare variants and might be underpowered to identify respective associations. It is further not large enough to conduct analyses stratified by sex, menopausal status, or aetiology of osteoporosis. Analyses in stratified, less heterogeneous samples, generally have a higher statistical power and yield more precise effect estimates due to the reduction of data variability. This advantage is, however, outweighed by the reduction of sample size, which substantially decreases statistical power. Despite these limitations, our analyses among the heterogeneous patient cohorts generally support the results from the genome-wide association study. This, in turn, strongly argues for the robustness of our results. The genetic effects obtained from the population-based cohorts assuming a commonly used additive genetic association model were reflected in MAF differences when compared to the patient cohorts, although the analyses were underpowered to reach statistical significance. Furthermore, we could not distinguish between dominant or recessive effects.

## Conclusion

Taken together, our results confirm 45 genetic variants to be associated with ultrasound-based heel stiffness index in adult men and women from the general population. Among these, two non-coding variants in the *WNT16* locus (rs2707518, rs3779381) as well as a novel, possibly causal association of rs603424 mediated via *SCD*, were demonstrated to impact the examined bone parameter. Our results, thus, highlight the effect of the Wnt-16 pathway in the regulation of bone properties and indicate a role of *SCD* expression in adipose tissue on bone substance.

### Supplementary Information

Below is the link to the electronic supplementary material.

Supplementary file1 (TIF 84884 kb)

Supplementary file2 (DOCX 23 kb)

Supplementary file3 (XLSX 484 kb)
