# Supplementary material for: Genetic Variants in WNT16 and PKD2L1 Locus Affect Heel Ultrasound Bone Stiffness: Analyses from the General Population and Patients Evaluated for Osteoporosis
Source: Calcif Tissue Int. 2023 Oct 13;113(5):540–51. doi: 10.1007/s00223-023-01141-9 (PMC10618371; doi:10.1007/s00223-023-01141-9)
Supplement: Supplementary file 2 — Supplementary file2 (DOCX 23 kb) [file 223_2023_1141_MOESM2_ESM.docx]

**Supplemental Table 1**. Associations of the selected SNPs with spine and femoral neck BMD in the patients evaluated for osteoporosis. Results from linear regression models adjusted for sex and age.

| **HSD** | | | | | | | | | | | |
| --- | --- | --- | --- | --- | --- | --- | --- | --- | --- | --- | --- |
| **dbSNP ID** | **Position** | **Genetic variants** | **Genetic location** | **Spine** | | | | **Femoral neck** | | | |
|  |  |  |  | **Effect size** | **Stderr** | **p** | **n** | **Effect size** | **Stderr** | **p** | **n** |
| rs2707518 | chr7:120954908 | G>T | *CPED1/WNT16* | -0.007 | 0.012 | 0.532 | 376 | 0.001 | 0.008 | 0.875 | 374 |
| rs3779381 | chr7:120966790 | A>G | *WNT16* | 0.002 | 0.014 | 0.904 | 380 | -0.009 | 0.009 | 0.330 | 378 |
| rs115242848 | chr2:119507607 | C>T | *LOC101927709/EN1* | 0.032 | 0.081 | 0.689 | 370 | -0.008 | 0.055 | 0.883 | 369 |
| rs10239787 | chr7:27970153 | C>T | *JAZF1* | 0.012 | 0.012 | 0.305 | 377 | 0.011 | 0.008 | 0.169 | 375 |
| rs6968704 | chr7:27966984 | C>T | *JAZF1* | 0.001 | 0.013 | 0.934 | 379 | 0.000 | 0.009 | 0.971 | 377 |
| rs603424 | chr10:102075479 | G>A | *PKD2L1* | -0.009 | 0.013 | 0.484 | 379 | -0.008 | 0.009 | 0.412 | 377 |
| **OsteoGene** | | | | | | | | | | | |
| **dbSNP ID** | **Position** | **Genetic variants** | **Genetic location** | **Spine** | | | | **Femoral neck** | | | |
|  |  |  |  | **Effect size** | **Stderr** | **p** | **n** | **Effect size** | **Stderr** | **p** | **n** |
| rs2707518 | chr7:120954908 | G>T | *CPED1/WNT16* | 0.003 | 0.015 | 0.854 | 230 | 0.002 | 0.011 | 0.822 | 228 |
| rs3779381 | chr7:120966790 | A>G | *WNT16* | 0.005 | 0.016 | 0.737 | 230 | -0.002 | 0.012 | 0.846 | 228 |
| rs115242848 | chr2:119507607 | C>T | *LOC101927709/EN1* | 0.063 | 0.110 | 0.567 | 227 | 0.011 | 0.081 | 0.891 | 225 |
| rs10239787 | chr7:27970153 | C>T | *JAZF1* | -0.008 | 0.015 | 0.602 | 228 | 0.007 | 0.011 | 0.522 | 226 |
| rs6968704 | chr7:27966984 | C>T | *JAZF1* | 0.013 | 0.016 | 0.422 | 230 | -0.003 | 0.012 | 0.834 | 228 |
| rs603424 | chr10:102075479 | G>A | *PKD2L1* | -0.007 | 0.019 | 0.694 | 230 | -0.002 | 0.014 | 0.878 | 228 |

| **Patients evaluated for osteoporosis (combined HSD and OsteoGene)** | | | | | | | | | | | |
| --- | --- | --- | --- | --- | --- | --- | --- | --- | --- | --- | --- |
| **dbSNP ID** | **Position** | **Genetic variants** | **Genetic location** | **Spine** | | | | **Femoral neck** | | | |
|  |  |  |  | **Effect size** | **Stderr** | **p** | **n** | **Effect size** | **Stderr** | **p** | **n** |
| rs2707518 | chr7:120954908 | G>T | *CPED1/WNT16* | -0.004 | 0.010 | 0.654 | 606 | 0.002 | 0.007 | 0.779 | 602 |
| rs3779381 | chr7:120966790 | A>G | *WNT16* | 0.005 | 0.011 | 0.622 | 610 | -0.005 | 0.008 | 0.562 | 606 |
| rs115242848 | chr2:119507607 | C>T | *LOC101927709/EN1* | 0.036 | 0.068 | 0.596 | 597 | -0.004 | 0.049 | 0.931 | 594 |
| rs10239787 | chr7:27970153 | C>T | *JAZF1* | 0.005 | 0.010 | 0.604 | 605 | 0.010 | 0.007 | 0.149 | 601 |
| rs6968704 | chr7:27966984 | C>T | *JAZF1* | 0.003 | 0.010 | 0.787 | 609 | -0.004 | 0.008 | 0.639 | 605 |
| rs603424 | chr10:102075479 | G>A | *PKD2L1* | -0.012 | 0.011 | 0.307 | 609 | -0.007 | 0.008 | 0.372 | 605 |

Position based on GRCh37.p13; Variation, major>minor allele, in the regression analysis the minor allele was the coded allele; Effect size, unstandardized regression coefficient; Stderr, standard error; p, significance; n, number of patients included in the analysis

**Supplemental Table 2.** Comparison of the MAF in the SHIP participants and in the patients evaluated for osteoporosis. We hypothesized that a minor allele with protective effect on BMD (as inferred from the meta-analysis) is less represented in the patients evaluated for osteoporosis whereas a minor allele with negative effect on BMD is more represented.

| **dbSNP ID** | **Gene** | **Effect SHIP** | **MAF SHIP** | **MAF HSD** | **Diff HSD-SHIP** | **Conf HSD** | **MAF OsteoGene** | **Diff OsteoGene-SHIP** | **Conf OsteoGene** | **MAF Patients** | **Diff Patients-SHIP** | **Conf Patients** |
| --- | --- | --- | --- | --- | --- | --- | --- | --- | --- | --- | --- | --- |
| rs2707518 | *CPED1/WNT16* | 2.327 | 0.372 | 0.374 | 0.002 | no | 0.353 | -0.018 | yes | 0.366 | -0.005 | yes |
| rs3779381 | *WNT16* | 2.229 | 0.240 | 0.223 | -0.017 | yes | 0.222 | -0.018 | yes | 0.223 | -0.018 | yes |
| rs115242848 | *LOC101927709/EN1* | 6.702 | 0.013 | 0.005 | -0.008 | yes | 0.004 | -0.009 | yes | 0.005 | -0.008 | yes |
| rs10239787 | *JAZF1* | -1.587 | 0.358 | 0.353 | -0.005 | no | 0.357 | -0.001 | no | 0.354 | -0.004 | no |
| rs6968704 | *JAZF1* | 1.427 | 0.302 | 0.302 | 0.000 | no | 0.291 | -0.011 | yes | 0.298 | -0.004 | yes |
| rs603424 | *PKD2L1* | -1.956 | 0.178 | 0.201 | 0.023 | yes | 0.188 | 0.009 | yes | 0.196 | 0.018 | yes |

Effect, unstandardized regression coefficient; MAF, minor allele frequency; Diff, difference of MAF of the studies; Conf, confirmation of hypothesis

**Supplemental Table 3.** Results of the Bayesian co-localization analysis (see Excel File)

**Supplemental Table 4.** SMR results (see Excel file)

**Supplemental Table 5.** Associations between rs603424 and the abdominal subcutaneous (SAT) and visceral (VAT) adipose tissue and the ratio of VAT / SAT. Results from linear regression models.

| **Outcome** | **SHIP-START-2 (n = 951)** | | |  | **SHIP-TREND-0 (n = 1,803)** | | |  | **Meta-analysis** | | |
| --- | --- | --- | --- | --- | --- | --- | --- | --- | --- | --- | --- |
|  | **Effect Size** | **Stderr** | **p** |  | **Effect Size** | **Stderr** | **p** |  | **Effect Size** | **Stderr** | **p** |
| **SAT, l** | 0.039 | 0.024 | 0.102 |  | -0.014 | 0.019 | 0.455 |  | 0.006 | 0.015 | 0.667 |
| **VAT, l** | 0.028 | 0.034 | 0.402 |  | 0.001 | 0.027 | 0.967 |  | 0.011 | 0.021 | 0.581 |
| **VAT / SAT** | -0.011 | 0.021 | 0.595 |  | 0.015 | 0.015 | 0.322 |  | 0.006 | 0.012 | 0.633 |

All models were adjusted for sex and age. As genotyping in the SHIP-TREND cohort was performed with two arrays, an additional adjustment for batch and the first two principle components was performed. SAT, VAT and the ratio of VAT / SAT were log-transformed. A fixed effects inverse-variance weighted meta-analysis was performed to combine the results of SHIP-START-2 and SHIP-TREND-0. Effect size, unstandardized regression coefficient; Stderr, standard error; p, significance; n, number of patients included in the analysis

**Supplemental Table 6.** Known or proposed cellular functions of the selected genes. Information on genes was obtained from Ensembl, Release 110 (July 2023) (<https://www.ensembl.org/index.html>, accessed September 10, 2023). Information on encoded proteins and functions were obtained from Uniprot, Release 2023_03 (<https://www.uniprot.org/>, accessed September 10, 2023).

| **Gene** | **Ensembl ID** | **Full name** | **Encoded protein** | **UniProt ID** | **Function** |
| --- | --- | --- | --- | --- | --- |
| *WNT16* | ENSG00000002745 | Wnt family member 16 | Wnt-16 | Q9UBV4 | development (probably) |
| *EN1* | ENSG00000163064 | engrailed homeobox 1 | homeobox protein engrailed-1 | Q05925 | development (formation of apical ectodermal ridge, correct dorsal-ventral patterning in the limb) |
| *JAZF1* | ENSG00000153814 | JAZF zinc finger 1 | juxtaposed with another zinc finger protein 1 | Q86VZ6 | transcriptional corepressor of NR2C2;  gluconeogenesis, lipid metabolism and glucose homeostasis |
| *PKD2L1* | ENSG00000107593 | polycystin 2 like 1, transient receptor potential cation channel | polycystin-2-like protein 1 | Q9P0L9 | subunit of a cation channel (Ca^2+^); calcium concentration in primary cilia, sonic hedgehog/SHH signaling in primary cilia, sour taste perception |
| *SCD* | ENSG00000099194 | stearoyl-CoA desaturase | stearoyl-CoA desaturase | O00767 | lipid biosynthesis |
